# Signal in Space Error and Ephemeris Validity Time Evaluation of Milena and Doresa Galileo Satellites †

**DOI:** 10.3390/s19081786

**Published:** 2019-04-14

**Authors:** Umberto Robustelli, Guido Benassai, Giovanni Pugliano

**Affiliations:** Department of Engineering, Parthenope University of Naples, 80143 Napoli, Italy; guido.benassai@uniparthenope.it (G.B.); giovanni.pugliano@uniparthenope.it (G.P.)

**Keywords:** Galileo, Doresa, Milena, broadcast ephemeris, precise ephemeris, sp3, SISE Signal In Space Error, broadcast ephemeris validity time

## Abstract

In August 2016, Milena (E14) and Doresa (E18) satellites started to broadcast ephemeris in navigation message for testing purposes. If these satellites could be used, an improvement in the position accuracy would be achieved. A small error in the ephemeris would impact the accuracy of positioning up to ±2.5 m, thus orbit error must be assessed. The ephemeris quality was evaluated by calculating the SISE_orbit_ (in orbit Signal In Space Error) using six different ephemeris validity time thresholds (14,400 s, 10,800 s, 7200 s, 3600 s, 1800 s, and 900 s). Two different periods of 2018 were analyzed by using IGS products: DOYs 52–71 and DOYs 172–191. For the first period, two different types of ephemeris were used: those received in IGS YEL2 station and the BRDM ones. Milena (E14) and Doresa (E18) satellites show a higher SISE_orbit_ than the others. If validity time is reduced, the SISE_orbit_ RMS of Milena (E14) and Doresa (E18) greatly decreases differently from the other satellites, for which the improvement, although present, is small. Milena (E14) and Doresa (E18) reach a SISE_orbit_ RMS of about 1 m (comparable to that of the other Galileo satellites reach with the nominal validity time) when validity time of 1800 s is used. Therefore, using this threshold, the two satellites could be used to improve single point positioning accuracy.

## 1. Introduction

On 15 December 2016, with 18 satellites in orbit, Europe’s satellite navigation system Galileo was declared operational and started offering its initial services to public authorities, businesses and citizens. When Galileo is fully operational, the constellation will consist of 24 satellites plus spares in Medium Earth Orbit (MEO) at an altitude of 23,222 km. Eight active satellites will occupy each of three orbital planes inclined at an angle of 56${}^{\circ }$ to the equator. The satellites will be spread evenly around each plane and will take about 14 h to orbit the Earth. Two further satellites in each plane will be a spare on standby should any operational satellite fail.

Currently, (February 2019), Galileo is in its full operational capability (FOC) phase; 22 FOC satellites were launched up to the start of 2019 in addition to the four IOV launched between 2011 and 2012. The navigation signals of these satellites are transmitted on five frequencies: E1, E5a, E5b, E5 and E6. Only three IOV satellites are operational because E20 has been declared unavailable since 27 May 2014 [1] when a power anomaly led E5 and E6 signals to a permanent loss of power. After this failure, all IOV satellites are backed-off and thus their signals have less transmitted power than the FOC satellites. Nineteen FOC satellites are declared operational, one (E22) was removed from active service in December 2017 for constellation management purposes, while the first two FOC satellites, Milena (E14) and Doresa (E18), result under test. These two satellites were launched on 22 August 2014 at 09:27 local time in French Guiana by a Soyuz ST rocket. The two satellites were left in a non-nominal highly elliptical orbit characterized by an apogee of 25,900 km and a perigee of 13,713 km, with an inclination with respect to the equator of 49.69${}^{\circ }$ instead of planned circular medium-Earth orbits at an altitude of 23,222 km with an inclination of 55.04${}^{\circ }$. The wrong injection orbits made the two satellites not usable for navigation mission, thus ESA planned a salvaged mission to make the two satellites usable [2,3]. From November 2014 to February 2015, the satellites made a series of maneuvers to raise the low point of their orbits by 3500 km and make their orbits more circular. Satellites in new orbits overfly the same location on the ground every 20 days. This is different from the nominal Galileo repeat pattern of 10 days, but makes possible a synchronization of theirs ground track with the rest of the Galileo constellation. Thus, the revised orbit (reported in Table 1) allowed ESA to switched on satellite’s navigation payload. On 5 August 2016, beginning 00:00 UTC, GSAT0201 named Doresa (E18) and GSAT0202 named Milena (E14) started broadcasting navigation message for testing purposes. (For details, see notice advisory to Galileo users https://www.gsc-europa.eu/notice-advisory-to-galileo-users-nagu-2016030).

At the end of the mission, the European Space Agency on 8 October 2016 requested to provide feedback on usage of these satellites (http://galileognss.eu/tag/sat-6/). After the ESA request, Sosnica et al. [4] in 2016 analyzed the accuracy of the Galileo system orbits including Milena (E14) and Doresa (E18) satellites, focusing on the precise orbits. Giorgi et al. [5] tested the general relativity studying the relativistic offset (redshift and Doppler shift) by using data provided by E18 Galileo satellite signals. In 2018, Paziewski et al. [6] investigated the potential use of Milena (E14) and Doresa (E18) satellites for positioning purposes. They evaluated the applicability of the Milena (E14) and Doresa (E18) satellites to precise GNSS positioning focusing on relative kinematic positioning as an instantaneous solution in multi-baseline mode. Robustelli and Pugliano [7] analyzed the code multipath error of GNSS satellites by using the short time Fourier transform and Wavelet analysis [8], showing that multipath performance of Milena (E14) and Doresa (E18) satellites are the same as the other Galileo FOC satellites when a mobile phone is used as receiver [9]. In 2018, Nicolini and Caporali [10] analyzed Milena (E14) and Doresa (E18) satellites’ orbits during Week 1950 from 21 May 2017 to 27 May 2017. The Galileo system is constantly evolving and the dataset analyzed by Nicolini and Caporali does not fully cover the ground track of the satellites, thus a re-evaluation is justified. Moreover, they considered a period of validity of the Galileo ephemeris equal to 1 h, less than that fixed by the ESA of 4 h.

**Figure 1:**
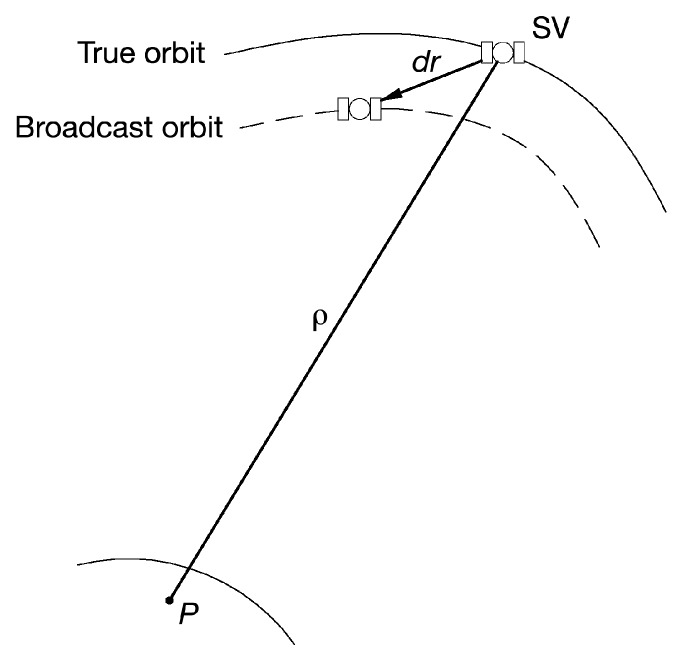
Ephemeris error: *dr* is the radial residual satellite position error, ρ is the pseudorange measurement and *P* is the point where receiver is located.

GNSS errors can be divided into errors that can be corrected by using differential techniques or not. The multipath error belongs to first group [11] while the ephemeris error to the second. GNSS satellites travel in precise and well known orbits. Unfortunately, the orbits have slight variations that lead to significant errors in the calculated positions. For these reasons, the GNSS ground control system continually monitors the satellite orbit. In the event that the orbit of a satellite changes, the ground control system sends a message to the satellite through which the content of the broadcast ephemeris is updated. However, even with the corrections from the GNSS ground control system, there are still small errors in the orbit that can result in up to ±2.5 m of position error [12]. The ephemeris errors are the differences between the true satellite position and the position computed using the GNSS navigation message. The radial residual satellite position error (*dr*) is a vector that is depicted in Figure 1 where ρ represents the pseudorange measurement and *P* the point where receiver is located. The typical magnitudes of ephemeris error is in the range of 1–6 m [13]. To make the Milena (E14) and Doresa (E18) satellites usable, it is necessary that the broadcast ephemeris must have an accuracy comparable to that of the other Galileo satellites. Actually, it is very important to have two additional satellites available since currently Galileo system is not yet fully operational and the accuracy in the position increases as the number of visible satellites increases. We are focusing our attention on the broadcast ephemeris because they are the only ones that can be used in real time for single point positioning, a lack of accuracy on them will directly impact the accuracy of Galileo positioning. For this reason, the evaluation of the accuracy of the ephemeris broadcasted by the Milena (E14) and Doresa (E18) satellites is of fundamental importance and is the main purpose of this paper. In a first analysis conducted by the authors [14], the position error between the broadcast orbit and the IGS precise orbit was determined for each second for the period starting from 21 February (DOY 52) to 12 March (DOY 71) of 2018 by using ephemeris decoded by receiver located YEL2 station. The analysis showed that the orbits of the satellites Milena (E14) and Doresa (E18) are affected by an error comparable to that of the other Galileo satellites with the exception of DOY 62 during which two anomalies were found. The two anomalies were located in the time range between 528,000 and 531,000 s and between 556,000 and 559,000 s for Milena (E14) and Doresa (E18) satellites, respectively.

This study investigated these anomalies by comparing the position of each Galileo satellite obtained by MGEX multi-GNSS broadcast ephemeris (BRDM) with precise orbit every second for the above mentioned dataset and for an additional twenty-day dataset from 21 June 2018 (DOY 172) to 10 July 2018 (DOY 191). The BRDM ephemeris are different from received one: they are generated by Technische Universitat Munchen (TUM) and DLR by merging real-time streams of a number of selected MGEX stations. For the first period (DOY 52–71), two different types of transmitted ephemerides were used: those received by the receiver placed in IGS YEL2 station located in Canada and the BRDM ones. During the first period, 16 satellites were working while during the second there were 19 satellites.

## 2. Materials and Methods

### 2.1. Methodology

Galileo broadcast ephemeris contains Keplerian elements to describe the motion of satellite. Galileo system broadcast four different navigation messages: free accessible navigation (F/NAV) provided by E5a-I signal for open service, integrity navigation (I/NAV) provided by E5b and E1-B signal corresponding to both open and commercial service, Commercial Navigation Message C/NAV provided by E6-B signal supporting commercial service and the Governmental Navigation Message (G/NAV) provide by E1A and E6A signals. Currently, only F/NAV and I/NAV are available since the G/NAV navigation message does not belong to the public domain and the C/NAV is not yet defined.

Since the Keplerian parameters transmitted in the two messages are the same, in this work, we indifferently used F/NAV or I/NAV messages paying attention to use the one that has a minor age.

The Signal In Space Error (SISE) is considered a key performance indicator of all navigation systems. It provides the instantaneous difference between the Galileo satellite position/clock offset as obtained from the broadcast Navigation message and the “true” satellite position/clock offset. It can be used to assess the quality of clock and ephemeris contained in the navigation message transmitted by Galileo satellites. It is a function of time and user location within the satellite coverage area. SISE is defined as the difference of the satellite position and time as broadcast by the navigation message and the true satellite position and time, projected on the user-satellite direction [15]. It can be computed by comparing the predicted satellite position and time, based on the broadcast navigation message, with a posteriori precise clock and orbit estimations. Its analytic expression can be derived from the Galileo satellite’s orbit error components expressed in along-track (A), cross-track (C) and radial (R) frame and its total Signal In Space (SIS) clock prediction error (CLK) by means of the following formula.

(1) $$SISE=\sqrt{0.9673\cdot R^{2}+CLK^{2}+0.01632\cdot \left(A^{2}+C^{2}\right)+1.967\cdot CLK\cdot R}$$

**Figure 2:**
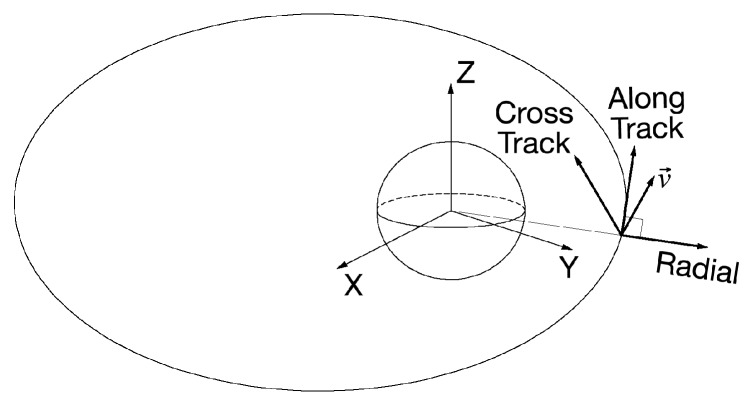
Along-track (A), cross-track (C) and radial (R).

The ACR coordinate system is depicted in Figure 2. Its origin is located at the centroid of the space object. The radial axis always points from the Earth’s center along the radius vector toward the satellite as it moves through the orbit; the along-track axis (or transverse) points in the direction of (but not necessarily parallel to) the velocity vector and is perpendicular to the radius vector; the cross-track axis is normal to the plane identified by velocity and radius vectors [16].

We evaluated the accuracy of Milena (E14) and Doresa (E18) Galileo satellites broadcasted ephemeris by using the so-called in orbit SISE (SISE_orbit_). It is derived by SISE without considering the clock error thus considering CLK = 0 in Equation (1). SISE_orbit_ can be expressed as follows:

(2) $$SISE_{orbit}=\sqrt{0.9673\cdot R^{2}+0.01632\cdot \left(A^{2}+C^{2}\right)}$$

To calculate SISE_orbit_, we used as reference the IGS precise orbits. They are generally more accurate than the broadcast orbits by almost two orders of magnitude [17].

Thus, we assumed that the precise orbits could be considered the truth and any difference between the two is attributed to broadcast orbit error.

The first step was to determine the position and velocity of the Galileo satellites at a certain time instant by the propagation of the Keplerian parameters contained in broadcast ephemeris using the algorithm described in Galileo Navigation Signal in Space Interface Control Document (ICD) [18]. At the end of this stage, we had satellites position and velocity expressed in the Earth Centered Earth Fixed (ECEF) frame. As recommended in European GNSS (Galileo) quarterly performance report [19], we performed a quality check on transmitted messages discarding parameters relative to satellites whose Signal Health Status (SHS) bits are set to 01 or to 10 (see Table 2) or whose age is beyond ephemeris validity time (VT) that is the maximum usability period of broadcasted navigation parameters. It should be emphasized that we did not discard the satellites with the SHS parameter set to “currently under test; because for the Milena (E14) and Doresa (E18) satellites SHS were set to “Test” and the Data Validity Status (DVS) flags to WWG (working without guarantee) since 8 October 2016 (https://galileognss.eu/galileo-satellites-in-eliptical-orbit-broadcasting-navigation-messages/).

Currently, VT is 4 h as reported in [19,20]. The age of ephemeris were estimated according to methodology described in Annex B “estimating the age of ephemeris” [20] as follow:

(3) $$t_{aoe}=t_{tom}-t_{oe}$$

where $t_{aoe}$ is the age of ephemeris, $t_{tom}$ is the transmission time of the navigation message and $t_{oe}$ is the time when ephemeris parameters had been calculated. To determine if the navigation parameters can be used, the value of $t_{aoe}$ must satisfy the following inequality:

(4) $$0\leq t_{aoe}\leq VT$$

Thus, the ephemerides that do not respect the inequality in Equation (4) were discarded. The next step consisted in the determination of the position and the velocity of satellites for each epoch starting from precise orbit parameters. The sp3 format stores the ECEF coordinates and velocities of all satellites every 900 s in the IGS14 frame, a frame adopted by IGS in order to align IGS products to International Terrestrial Reference Frame 2014 (ITRF2014) frame [21].

The satellites coordinates, at epoch t were obtained by interpolating parameters contained in sp3 format with a Lagrange polynomial function of 16th order in the time window [t − 2h, t + 2h] contained in the precise ephemeris sp3 files.

The satellite position in the broadcast ephemeris is referenced to the GTRF (Galileo Terrestrial Reference Frame) while the precise ephemeris is provided in the IGS14 frame aligned with the ITRF2014. According to Cai et al. [22], the difference in the two coordinate systems is only about 1–3 cm, thus the Galileo broadcast reference frame can be considered aligned with ITRF2014 as already done Nicolini and Caporali [10].

It is important to underline that the broadcast orbits are determined with respect to the satellite’s antenna phase center while the precise orbits are relative to satellite’s center of mass. Thus, the coordinates obtained by broadcasted ephemeris must be referred to the center of mass [23] using the correction values calculated in [24] and reported in Table 3.

Now, the difference between the satellites positions obtained starting from broadcast and precise orbits can be calculated and expressed in ECEF frame as:

(5) $$\Delta P=\left[\begin{array}{c} X^{brdc}-X^{pre}\\ Y^{brdc}-Y^{pre}\\ Z^{brdc}-Z^{pre} \end{array}\right]$$

(6) $$\Delta V=\left[\begin{array}{c} V_{X}^{brdc}-V_{X}^{pre}\\ V_{Y}^{brdc}-V_{Y}^{pre}\\ V_{Z}^{brdc}-V_{Z}^{pre} \end{array}\right]$$

where the brdc and pre superscripts indicate that the coordinates and velocities were obtained starting from the transmitted and precise ephemeris, respectively.

The errors reported in Equations (5) and (6) (referred to a rotating system) are the same as in an inertial system since the inertial and rotating coordinates differ for an additive term, thus, after calculating the unit vectors of the three axes of ACR coordinate system in ECI frame according to these relations:

(7) $${\hat{R}}_{ECI}=\frac{{\vec{r}}_{sat}^{ECI}}{∥{\vec{r}}_{sat}^{ECI}∥};\;{\hat{C}}_{ECI}=\frac{{\vec{r}}_{sat}^{ECI}\times {\vec{v}}_{sat}^{ECI}}{∥{\vec{r}}_{sat}^{ECI}\times {\vec{v}}_{sat}^{ECI}∥};\;{\hat{A}}_{ECI}={\hat{C}}_{ECI}\times {\hat{R}}_{ECI};$$

we can express ΔP in ACR frame by using:

(8) $$\left[\begin{array}{c} \Delta P_{radial}\\ \Delta P_{along}\\ \Delta P_{cross} \end{array}\right]=\left[\begin{array}{ccc} {\hat{R}}_{X}^{ECI} & {\hat{R}}_{Y}^{ECI} & {\hat{R}}_{Z}^{ECI}\\ {\hat{A}}_{X}^{ECI} & {\hat{A}}_{Y}^{ECI} & {\hat{A}}_{Z}^{ECI}\\ {\hat{C}}_{X}^{ECI} & {\hat{C}}_{Y}^{ECI} & {\hat{C}}_{Z}^{ECI} \end{array}\right]\left[\begin{array}{c} \Delta P_{X}^{ECI}\\ \Delta P_{Y}^{ECI}\\ \Delta P_{Z}^{ECI} \end{array}\right]$$

where $\Delta P_{X}^{ECI}$, $\Delta P_{Y}^{ECI}$ and $\Delta P_{Z}^{ECI}$ are the difference between the satellites coordinates obtained from the transmitted ephemeris and those obtained from the precise ephemeris along the X, Y and Z axes of the ECI frame, respectively. We developed a suitable software tool in MATLAB^®^ environment able to compute all Galileo satellites orbit error in ACR frame and relative SISE_orbit_. The errors and SISE_orbit_ were determined every seconds for the entire study using six different ephemeris validity time thresholds: 14,400 s, 10,800 s, 7200 s, 3600 s, 1800 s and 900 s.

### 2.2. Experimental Setup

The experiment was carried out using data produced by International GNSS Service (IGS). It is a voluntary association made up of universities, space agencies and geodetic agencies whose mission is “to provide the highest-quality GNSS data and products in support of the terrestrial reference frame, Earth rotation, Earth observation and research, positioning, navigation and timing and other applications that benefit society” [25]. IGS products are generated at several analysis centers (ACs) with strict quality control and proper weighting, thus having the highest quality and internal consistency. Due to the growth of GNSS constellations with the advent of the European Galileo, the Chinese Beidou, the Japanese QZSS and the Indian IRNSS, in 2012 IGS started the Multi-GNSS Experiment (MGEX) whose objective is to promote the generation of dedicated multi-GNSS precise orbit and clock products and the development of advanced processing algorithms [26,27]. The ground track of the Galileo FOC satellites Milena (E14) and Doresa (E18) repeats every 20 sidereal days, and therefore Milena (E14) and Doresa (E18) satellites do not reach the whole range of elevations during one single day. Three different datasets were analyzed.

The first dataset consists of the ephemeris decoded by receiver placed in YEL2 IGS station in the days from 21 February 2018 (DOY 52) to 12 March 2018 (DOY 71). It is related only to satellites that are visible in that location, however the elaborations related to the this dataset (already discussed in [14]) have been redone in order to calculate the orbit error in the ACR reference system. The second dataset is composed by the BRDM ephemeris relative to the same time range as the first one. The third dataset consists of BRDM ephemeris ranging from 21 June 2018 (DOY 172) to 10 July (DOY 191) 2018.

Precise orbits were obtained from the IGS website (ftp://igs.ensg.ign.fr/pub/igs/products/mgex/) that maintains precise orbit records from 1992 through to the present. Data are stored in SP3 (Standard Product 3) version c format as compressed (zip) files. It is an ASCII file that contains information about the precise orbital data and the associated satellite clock corrections.

Broadcast ephemeris were obtained from the Federal Agency for Cartography and Geodesy (BKG) GNSS data center website (https://igs.bkg.bund.de/dataandproducts/rinexsearch) referred to YEL2 station located in Canada. It is a regional IGS data center that collects observational and navigational data from several operational centers and stations, maintains a local archive of the data received and provides online access to these data to the user community.

The merged, multi-GNSS broadcast ephemeris containing all the unique broadcast navigation messages for the day were downloaded from the MGEX campaign archive at CDDIS web-site ftp://ftp.cddis.eosdis.nasa.gov/gnss/data/campaign/mgex/daily/rinex3.

## 3. Results

The results relating to the first dataset have already been discussed in [14], and constitute a subset of the results obtained by processing the second dataset. Therefore, in this section, we report only the results obtained by analyzing the second and third datasets.

**Figure 3:**
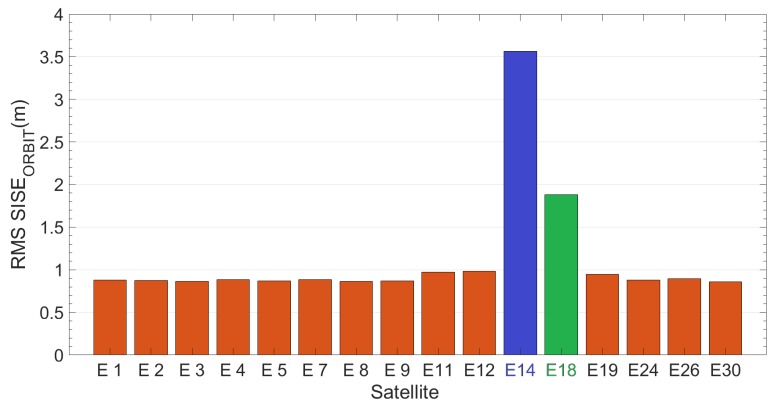
RMS SISE_orbit_ calculated on the dataset ranging from DOY 52 to 71 for all Galileo satellites. Blue and green are Milena (E14) and Doresa (E18) satellites, respectively. Ephemeris validity time threshold is set to 14,400 s.

Figure 3 reports Root-Mean-Square value of SISE_orbit_ calculated using an ephemeris validity time of 14,400 s (4 h) for each satellite. RMS has been calculated over time range of 20 days (DOYs 52–71). Milena (E14) and Doresa (E18) satellites (represented in figure in blue and green respectively) show a RMS of 3.56 and 1.88 m, respectively, which is four and two times the RMS shown by the other satellites (about 0.90 m).

The time evolution of the SISE_orbit_, radial, cross and along components of the two satellites are analyzed and compared with that of a satellite (E1) used as representative of all the others.

**Figure 4:**
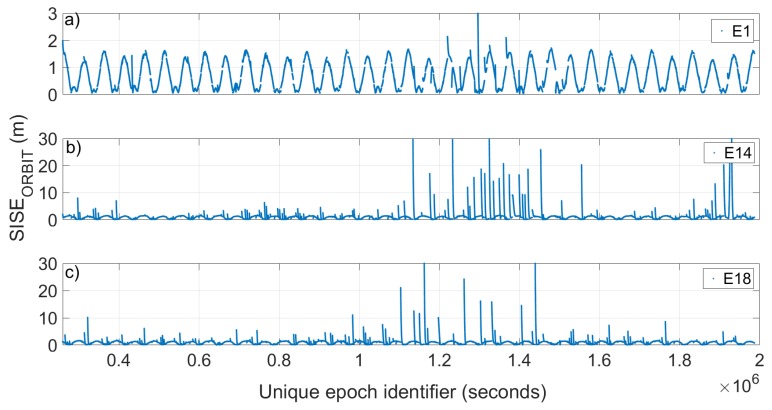
SISE_orbit_ versus time obtained with an ephemeris validity time of 14,400 s (4 h). Satellite 1 (E1), Milena (E14) and Doresa (E18) are plotted in (**a**–**c**), respectively. To have a better representation, in (**b**,**c**), the maximum value of the SISE_orbit_ shown is 30 m.

**Figure 5:**
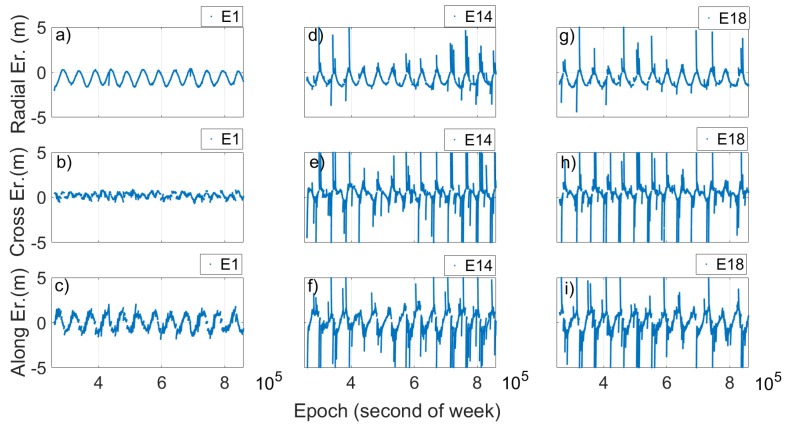
Radial, cross and along error for satellite 1 (E1), Milena (E14) and Doresa (E18) satellites plotted versus time. The ephemeris validity time is 14,400 s (4 h). To have a better readability for (**d**–**i**), the maximum value of errors is limited to 5 m.

Figure 4 shows the evolution of the SISE_orbit_ calculated with a period of validity of the ephemeris of 14,400 s (4 h) with respect to time for the satellites E1, Milena (E14) and Doresa (E18) reported in Figure 4a–c, respectively. Since 20 days of data are represented, it was not possible to use the Galileo epoch as univocal epoch because it is reset every new week. Thus, we used an epoch obtained by adding to its representation in Galileo time a number of seconds that takes into account the change of week. The E1 satellite was chosen as representative of all other Galileo satellites because they show very similar behaviour towards the orbit error. Figure 5 reports the radial, cross and along error components.

Looking at Figure 4 and Figure 5, two things are evident. The first is the extreme variation shown by SISE_orbit_ in the case of the Milena (E14) and Doresa (E18) satellites; the second is related to the clear periodicity that the SISE_orbit_ and error components show for all three satellites. Actually, Figure 4b,c shows a SISE_orbit_ up to 30 m, well below the real value, i.e. 236.1 and 49.5 m, respectively. Figure 5d–i shows zoomed in images to achieve a better readability. The maximum errors obtained are 7.8, 32.76, 11.34, 9.53, 29.08, and 12.24 m in Figure 5d–i, respectively.

**Figure 6:**
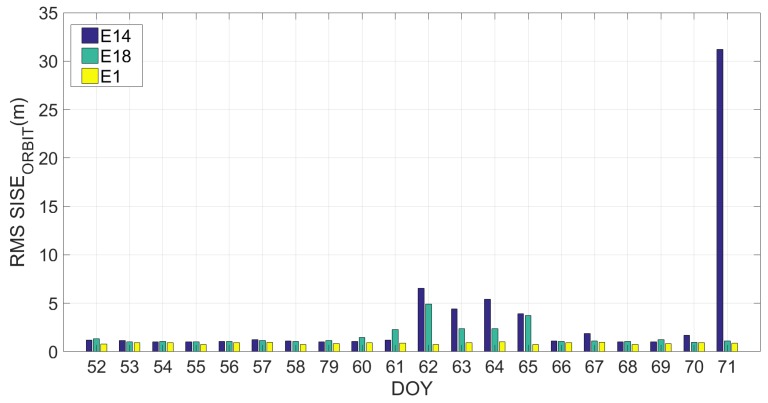
Daily RMS SISE_orbit_ calculated on the dataset ranging from DOY 52 to 71 for satellite 1 (E1), Milena (E14) and Doresa (E18) satellites reported in yellow, blue and green, respectively.

Figure 4 shows how the SISE_orbit_ varies with time. In particular, it can be noted that in some days it is very high while in others it is lower; therefore, instead of calculating an RMS of the SISE_orbit_ on the twenty days, a daily RMS was calculated, as reported in Figure 6. The figure shows clearly how the Milena (E14) and Doresa (E18) satellites have a higher SISE_orbit_ compared to the other satellites, with very high discrepancies on DOYs 61–65 and 71.

**Figure 7:**
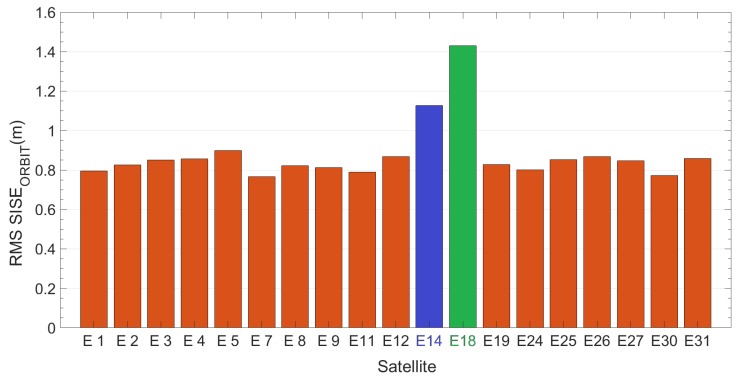
RMS SISE_orbit_ calculated on the dataset of DOY 172–191 for all Galileo satellites. Blue and green are Milena (E14) and Doresa (E18) satellites, respectively. Ephemeris validity time threshold is set to 14,400 s.

**Figure 8:**
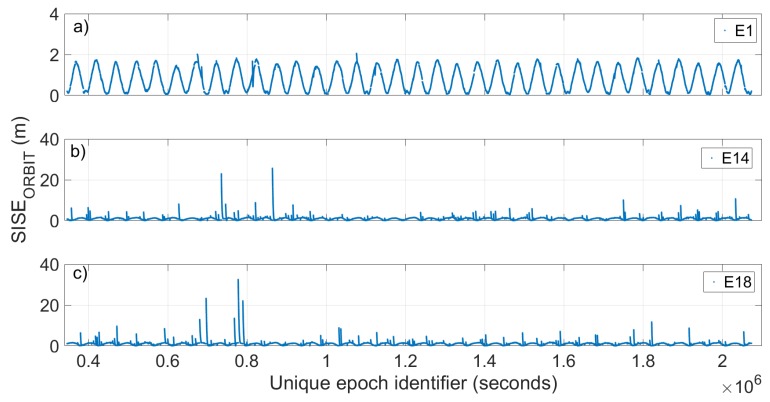
SISE_orbit_ versus time obtained with an ephemeris validity time of 14,400 s (4 h). Satellite 1 (E1), Milena (E14) and Doresa (E18) are plotted in (**a**–**c**), respectively. To have a better representation, in (**b**,**c**), the maximum value of the SISE_orbit_ shown is 3 m.

**Figure 9:**
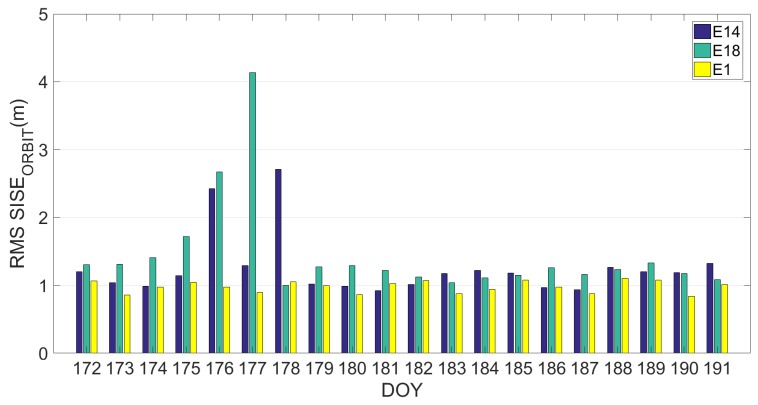
Daily RMS SISE_orbit_ calculated on the dataset of DOY 172–191 for satellite 1 (E1), Milena (E14) and Doresa (E18) satellites reported in yellow, blue and green respectively.

The same analysis was conducted on the third dataset. Results are reported in the Figure 7, Figure 8 and Figure 9. Figure 7 depicts the RMS value of SISE_orbit_ calculated using an ephemeris validity time of 14,400 s (4 h) for each satellite. Figure 8 shows the evolution of the SISE_orbit_ with respect to time for the satellites E1, Milena (E14) and Doresa (E18) reported in Figure 8a–c, respectively, while Figure 9 depicts the Daily RMS SISE_orbit_ for satellite Milena (E14) and Doresa (E18) compared with satellite E1.

If we compare Figure 3 and Figure 7, we see an improvement in SISE_orbit_: for satellite Milena (E14) the RMS is reduced from 3.56 to 1.12 m while for the Doresa (E18) satellite it decreases from 1.88 to 1.43 m. This improvement is also confirmed by what is shown in Figure 8 and Figure 9, nevertheless the SISE_orbit_ for the two satellites under analysis continues to be higher than that of the other Galileo satellites. Thus, this behaviour cannot have been caused by an anomaly because both analyzed datasets highlight it.

The validity time is a parameter that can be changed. Its reduction means using Keplerian parameter closer to the reference epoch and therefore the orbit error described by SISE_orbit_ decreases. However, the reduction of the validity time has a negative effect, as it will increase the number of epochs for which the parameters of the ephemeris will be too old, thus the satellite to which those parameters refer will be discarded in the navigation solution.

**Figure 10:**
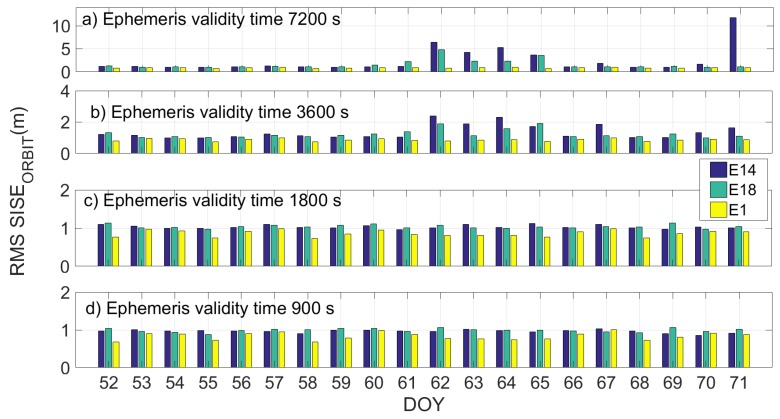
Daily RMS SISE_orbit_ for satellite Milena (E14) and Doresa (E18) and satellite 1 (E1) represented by blue, green and yellow bars, respectively. SISE_orbit_ was calculated using a validity time of 7200 s, 3600 s, 1800 s and 900 s in (**a**–**d**), respectively.

**Figure 11:**
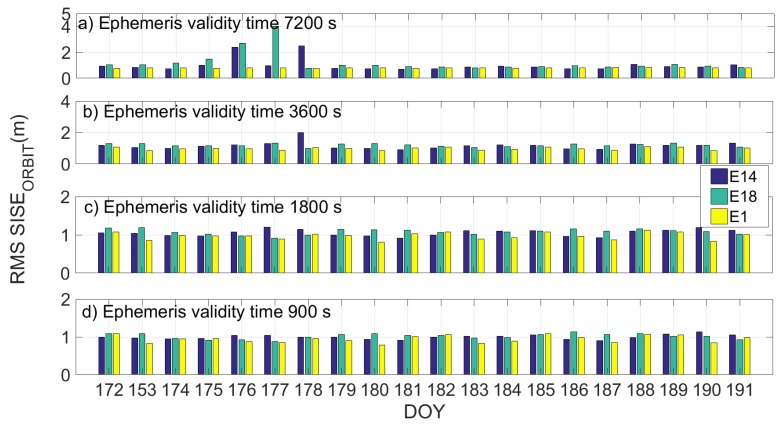
Daily RMS SISE_orbit_ for satellite Milena (E14) and Doresa (E18) and satellite 1 (E1) represented by blue, green and yellow bars, respectively. SISE_orbit_ was calculated by using a validity time of 7200 s, 3600 s, 1800 s and 900 s in (**a**–**d**), respectively. Figure refers to DOYs 172–191.

Figure 10 and Figure 11 show the daily RMS SISE_orbit_ calculated by using a validity time of 7200 s, 3600 s, 1800 s and 900 s in Figure 10a–d and Figure 11a–d, respectively, for the two different datasets. Milena (E14) satellite is represented by the blue bar, Doresa (E18) by the green one and satellite 1 (E1) represented by the yellow one. In the figures, it is evident how setting the ephemeris validity time equal to 1800 allows obtaining a SISE_orbit_ comparable with that of the other satellites.

The reduction in validity time has a positive effect on the Milena (E14) and Doresa (E18) satellites; observing the days with the higher RMS (DOYs 61–65, 71, and 175–178), we can see how this value decreases when the validity time decreases. This effect does not occur on the other satellites for which the improvement of the SISE_orbit_ with the diminution of the validity time is negligible. If a validity time of 1800 or 900 s is used, Milena (E14) and Doresa (E18) reach a SISE_orbit_ RMS of about 1 m comparable to that of the other Galileo satellites.

Table 4 reports SISE_orbit_ RMS value calculated on days with the higher RMS (DOYs 61–65, 71, and 175–178) using the six validity time thresholds.

**Figure 12:**
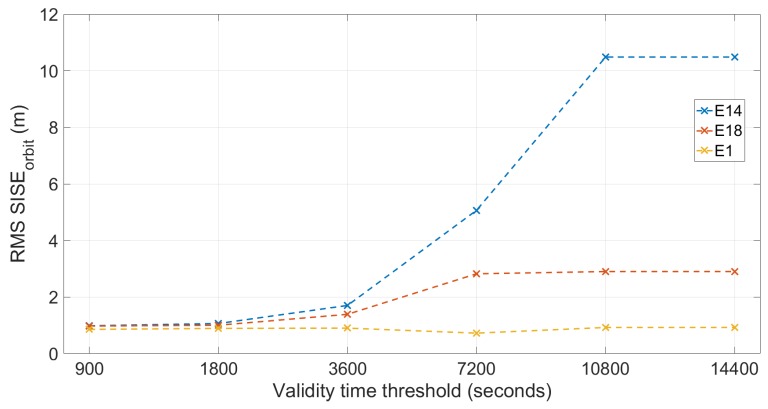
RMS SISE_orbit_ on DOYs with higher errors (DOYs 61–65, 71, and 175–178) plotted versus the validity time threshold used. Milena (E14) and Doresa (E18) and satellite 1 (E1) satellites are represented by blue, red and yellow lines, respectively.

Figure 12 shows the RMS SISE_orbit_ calculated on DOYs showing higher errors (DOYs 61–65, 71, and 175–178) plotted versus the six different validity time thresholds (values are reported in Table 4). By observing Figure 12, it can be seen that: the SISE of the satellite E1 does not change when the threshold used changes; for all three satellites, the errors with the threshold set at 14,400 s are identical to those obtained with the threshold set at 10,800 s, the errors of the Milena (E14) and Doresa (E18) satellites become comparable with those of the E1 satellite starting from the threshold of 3600 s; these errors become very similar when the thresholds are set at 1800 and 900 s. It should be highlighted that the use of such a low validity time has the consequence of discarding a certain number of epochs for which the validity of the message is below the threshold considered. This loss is calculated, as shown in Table 5 and Table 6 for the second and the third datasets, respectively.

Looking at the tables, we can see that the use of a threshold equal to 1800 s for validity time implies a loss of epochs equal to about 16% for Milena (E14) and about 11% for Doresa (E18) in the second analyzed dataset. Considering the third dataset, the discarded epochs decrease, passing to 5.78% for Milena (E14) and 8.3% for Doresa (E18). If a validity time of 900 s is used, the discarded epochs are about the 30% of the total. This led us to prefer a validity time of 1800 s compared to one of 900, since a small improvement in the error corresponds to a high reduction in usable periods. Therefore, considering a validity time of 1800 s, we could obtain a SISE_orbit_ comparable to that of the other satellites, but having to renounce the use of the two satellites in a number of epochs that is less than 8% in percentage terms. A validity time of 1800 s probably is better than one of 900 s because, by using the last one (as reported in Table 5 and Table 6), a higher number of epochs will be lost.

**Figure 13:**
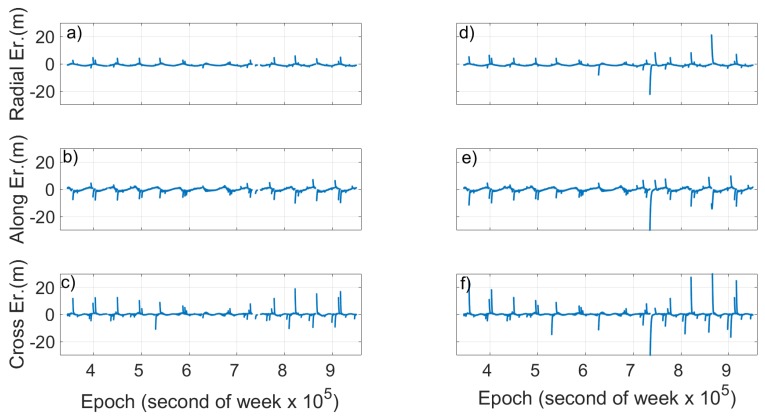
Comparison of radial, along and cross error for Milena (E14) obtained by using validity time threshold of: 1800 s (**left**); and 10,800 s (**right**). To have a better readability, the maximum value of errors is limited to 30 m. Errors that exceed this threshold relate to the cross component with a maximum error of 118.1 m (**f**). Figure refers to DOYs 172–191.

**Figure 14:**
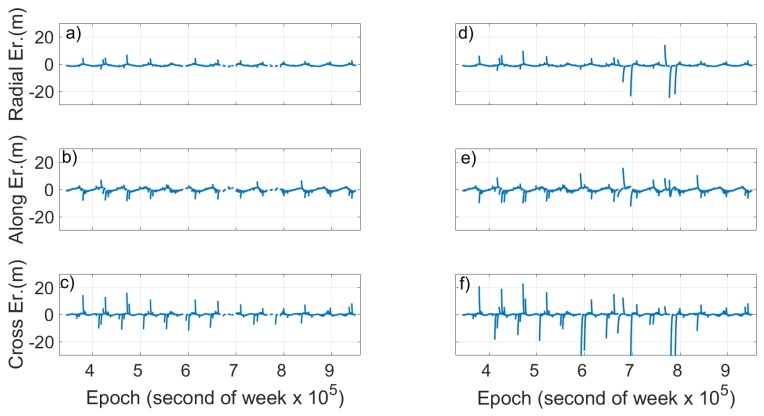
Comparison of radial, along and cross error for Doresa (E18) obtained by using validity time threshold of: 1800 s (**left**); and 10,800 s (**right**). To have a better readability, the maximum value of errors is limited to 30 m. Errors that exceed this threshold relate to the cross component with a maximum error of −173.1 m (**f**). Figure refers to DOYs 172–191.

Figure 13 and Figure 14 show the radial, cross and along errors calculated with the validity time threshold set at 1800 s (Figure 13 and Figure 14, left) and 10,800 s (Figure 13 and Figure 14, right) of Milena (E14) and Doresa (E18) satellites, respectively, for the second dataset of DOYs 172–191. By observing the two figures, an improvement of all three components can be noted when the validity time threshold is set at 1800 s. The radial component of a satellite’s ephemeris error is normally the smallest; however, it has the largest impact on the user’s calculated position. Along-track and cross-track components are larger than the radial component by an order of magnitude but have little impact of the resultant user position error [17]. Therefore, the improvement obtained using a threshold of 1800 s as validity time of ephemeris should allow us to be able to use the satellites under analysis as an augmentation to the navigational solution.

## 4. Discussion

The position error between the broadcast orbit and the IGS precise orbit was determined for each Galileo satellite every second using six different validity time ephemeris thresholds (14,400 s, 10,800 s, 7200 s, 3600 s, 1800 s, and 900 s) for two periods: the first starts on 21 February 2018 (DOY 52) and ends on 12 March 2018 (DOY 71), the second starts on 21 June 2018 (DOY 172) and ends on 10 July 2018 (DOY 191) by using IGS products. For the first period (DOYs 52–71), two different types of transmitted ephemeris were used: those received by the receiver located in IGS YEL2 station located in Canada and the brdm ones. During the first period, 16 satellites were working while during the second the satellites were 19, thus more than 2.2 × 10^8^ records were processed.

The analysis conducted show that Milena (E14) and Doresa (E18) satellites have a higher SISE_orbit_ than the other satellites of the Galileo constellation for all three datasets analyzed. Using a synthetic indicator such as the RMS calculated on the entire dataset the Milena (E14) satellite shows a RMS SISE_orbit_ equal to 3.56 m for the second dataset and of 1.12 m for the third dataset, while the Doresa (E18) satellite shows an RMS equal to 1.88 and 1.43 m for the same datasets. If the RMS calculation is done on a daily basis, the Milena (E14) and Doresa (E18) satellites have a higher RMS error with respect to the other satellites showing very high peaks in well-defined days (DOYs 61–65, 71, and 175–178).

Analyzing the temporal evolution of SISE_orbit_, we can see how Milena (E14) and Doresa (E18) satellites show discontinuities and very high peaks compared with those of the other Galileo satellites. Despite these differences, the SISE_orbit_ of Milena (E14) and Doresa (E18) satellites show the same periodicity of 13.9 h showed by the SISE_orbit_ of the other satellites, comparable with that was found by Nicolini and Caporali [10] for the other Galileo satellite. A big difference between Milena (E14) and Doresa (E18) with respect to the other satellites regards the validity time of their ephemeris: their orbital error shows a much higher sensitivity than the other satellites to the validity time variations. In particular, the reduction in validity time has a positive effect: by observing the days with the higher RMS (61–65, 71, and 175–178), it can be seen how this value decreases when the validity time decreases. This improvement is also highlighted in the radial, along and across components of the orbital error. As shown in the Results Section, to obtain a SISE_orbit_ comparable to that of the other satellites, it is necessary to decrease the validity time to 1800 s. This reduction involves a reduction of the epochs in which the satellites can be used. This reduction has been calculated in percentage terms: the epoch-loss is around 5% for Milena (E14) and 8% for Doresa (E18). This is a good result that must be verified appropriately in the position domain with single point positioning algorithms. Here, we want to recall that having two other satellites available will surely improve the achievable positioning accuracy. This aspect will be discussed in a forthcoming publication on which the authors are already working.

## Figures and Tables

**Table 1:** Galileo satellite nominal orbit parameter.

Satellite	Semi-Major Axis (Km)	Eccentricity (Km)	Inclination (deg)
IOV and FOC	29,599.8	0.000	56.0${}^{\circ }$
E14 and E18	27,977.6	0.162	49.850${}^{\circ }$

**Table 2:** Signal status bits definitions [18].

Signal Health Status Dec	Signal Health Status Bits	Definition
0	00	Signal OK
1	01	Signal out of service
2	10	Signal will be out of service
3	11	Signal Component currently in Test

**Table 3:** Antenna offsets for CoM correction of broadcast ephemeris [24].

Satellite Block	X(m)	Y(m)	Z(m)
IOV	+0.20	0.00	+0.60
FOC	−0.15	0.00	+1.00

**Table 4:** SISE_orbit_ RMS (m) in DOYs with higher errors (DOYs 61–65, 71, and 175–178) calculated at different validity time thresholds.

Sat	VT: 14,400 s	VT: 10,800 s	VT: 7200 s	VT: 3600 s	VT: 1800 s	VT: 900 s
E1	0.92	0.92	0.72	0.90	0.89	0.86
E14	10.48	10.48	5.06	1.70	1.06	0.98
E18	2.90	2.90	2.82	1.39	1.00	0.97

**Table 5:** Percentage of discarded epochs, DOYs 52–71.

Sat	VT: 14,400 s	VT: 10,800 s	VT: 7200 s	VT: 3600 s	VT: 1800 s	VT: 900 s
E1	0.04%	0.04%	0.32%	5.00%	15.83%	38.71%
E14	0.04%	0.04%	0.53%	5.35%	16.60%	37.82%
E18	0.04%	0.04%	0.04%	2.16%	11.36%	33.02%

**Table 6:** Percentage of discarded epochs, DOYs 172–191.

Sat	VT: 14,400 s	VT: 10,800 s	VT: 7200 s	VT: 3600 s	VT: 1800 s	VT: 900 s
E1	0.10%	0.10%	0.10%	0.79%	8.51%	33.44%
E14	0.10%	0.10%	0.10%	0.66%	5.78%	27.22%
E18	0.10%	0.10%	0.10%	1.67%	8.30%	29.58%
